# Answering Schrödinger's question: A free-energy formulation

**DOI:** 10.1016/j.plrev.2017.09.001

**Published:** 2018-03

**Authors:** Maxwell James Désormeau Ramstead, Paul Benjamin Badcock, Karl John Friston

**Affiliations:** a Department of Philosophy, McGill University, Montreal, Quebec, Canada; b Division of Social and Transcultural Psychiatry, Department of Psychiatry, McGill University, Montreal, Quebec, Canada; c Melbourne School of Psychological Sciences, The University of Melbourne, Melbourne, 3010, Australia; d Centre for Youth Mental Health, The University of Melbourne, Melbourne, 3052, Australia; e Orygen, the National Centre of Excellence in Youth Mental Health, Melbourne, 3052, Australia; f Wellcome Trust Centre for Neuroimaging, University College London, London, WC1N3BG, UK

**Keywords:** Free energy principle, Complex adaptive systems, Evolutionary systems theory, Hierarchically mechanistic mind, Physics of the mind, Variational neuroethology

## Abstract

The free-energy principle (FEP) is a formal model of neuronal processes that is widely recognised in neuroscience as a unifying theory of the brain and biobehaviour. More recently, however, it has been extended beyond the brain to explain the dynamics of living systems, and their unique capacity to avoid decay. The aim of this review is to synthesise these advances with a meta-theoretical ontology of biological systems called variational neuroethology, which integrates the FEP with Tinbergen's four research questions to explain biological systems across spatial and temporal scales. We exemplify this framework by applying it to Homo sapiens, before translating variational neuroethology into a systematic research heuristic that supplies the biological, cognitive, and social sciences with a computationally tractable guide to discovery.

## Highlights

• We describe a meta-theoretical ontology of life based on the free energy principle.
• We propose a multiscale formulation of the free energy principle.
• We translate our ontology into a systematic research heuristic for life sciences.
• We apply this meta-theoretical ontology and research heuristic to Homo sapiens.

## 1 Introduction

As Schrödinger [1] famously observed many years ago, living systems are unique among natural systems because they appear to resist the second law of thermodynamics by persisting as bounded, self-organizing systems over time. How is this remarkable feat possible? What is life? How is it realised in physical systems? And how can we explain and predict its various manifestations and behaviours? By asking such questions, Schrödinger inspired a new line of inquiry that broadly centres on evolutionary systems theory (EST), which has become one of the most pervasive paradigms in modern science. Closely related to complexity science, EST is an interdisciplinary field that builds on the pioneering efforts of theorists like Fisher, Wright, Haldane and Prigogine [2], [3], [4], [5], and has found expression in influential models such as Eigen and Schuster's hypercycles [6]. Put simply, EST explains dynamic, evolving systems in terms of the reciprocal relationship between general selection and self-organisation [7], [8], [9].

Originating from biology, general selection entails three interacting principles of change: variation, selection, and retention [10]. This Darwinian process not only applies to organisms (i.e., natural selection), but acts on all dynamically coupled systems (e.g., molecules, neural synapses, behaviours, theories, and technologies; [11], [12], [13]), and is a universal principle that cuts across both statistical and quantum mechanics [14], [15]. On the other hand, self-organisation stems from dynamic systems theory in physics [16], [17], [18], and refers to the emergence of functional, higher-order patterns resulting from recursive interactions among the simpler components of coupled dynamical systems over time (see Box 1). To date, research in EST has focused on complex adaptive systems—such as the brain [19], [20], social systems [21], and the biosphere [22]—which adapt to the environment through an autonomous process of selection that recruits the outcomes of locally interacting components—within that system—to select a subset for replication or enhancement [23].

At the turn of the millennium, the principles of EST inspired a new theory in neuroscience called the free-energy principle (FEP). Drawn chiefly from statistical thermodynamics and machine learning, the FEP is a formal model of neuronal processes that was initially proposed to explain perception, learning and action [24], [25], but has since been extended to explain the evolution, development, form, and function of the brain [26]. More recently, it has also been applied to biological systems across spatial and temporal scales, ranging from phenomena at the micro-scale (e.g., dendritic self-organisation and morphogenesis; [27], [28]), across intermediate scales (e.g., cultural ensembles; [29]), and at the macro-scale (e.g., natural selection; [30]). We believe that this theory puts us in a strong position to answer Schrödinger's question, and at the same time, shed new light on the mind, body, behaviour, and society.

We begin by describing how the FEP offers a plausible, mechanistic EST that applies to living systems in general. We then combine this variational principle with Tinbergen's four research questions in biology to describe a new scientific ontology—called variational neuroethology—that can be used to develop mathematically tractable, substantive explanations of living organisms. We conclude by applying this meta-theory to the most complex living system known to date—namely, ourselves—before translating this framework into a systematic research heuristic. In doing so, we hope to highlight a plausible, computationally tractable guide to discovery in the biological, cognitive and social sciences.

## 2 The free energy formulation

The FEP is a mathematical formulation that explains, from first principles, the characteristics of biological systems that are able to resist decay and persist over time. It rests on the idea that all biological systems instantiate a hierarchical generative model of the world that implicitly minimises its internal entropy by minimising free energy. In virtue of the self-organisation inherent in nonequilibrium steady-state, systems will apparently violate the second law of thermodynamics. See Ao et al. [31] for an interesting treatment of relative entropy in this context (that does not require detailed balance assumptions). From our perspective, this sort of behaviour can be cast in terms of a dynamics (i.e., conservative flow) that appears to minimise a variational free energy, which constitutes an upper bound on the entropy of a system's Markov blanket (see [32] and Box 2). Technically, free energy is an information theoretic quantity that limits (by being greater than) the entropy of sensory exchanges between a biotic system (e.g., the brain) and the environment. A generative model is a probabilistic mapping from causes in the environment to observed consequences (e.g., sensory data); while entropy refers to the (long-term) average of surprise (or surprisal)—the negative log probability of sensory samples encountered by an agent [26]. Under this formalism, for an organism to resist dissipation and persist as an adaptive system that is part of, coupled with, and yet statistically independent from, the larger system in which it is embedded, it must embody a probabilistic model of the statistical interdependencies and regularities of its environment. We elaborate on this next.

### 2.1 Living systems, ergodicity, and phenotypes

All biological systems exhibit a specific form of self-organisation, which has been sculpted by natural selection to allow them to actively maintain their integrity by revisiting characteristic states within well-defined bounds of their conceivable phase spaces (see Box 1). In other words, there is a high probability that an organism will occupy a relatively small, bounded set of states—its viability set [33]—within the total set of possible states that it might occupy (i.e., its phase space). In terms of information theory, this means that the probability density function that describes the possible states of the system has low entropy. So, how do living systems perform this feat?

This is simpler than it might seem, and rests on the fact that all living systems revisit a bounded set of states repeatedly (i.e., they are locally ergodic). At every scale—from the oscillations of neuronal activity over milliseconds, through to the pulsations of our heart and our daily routines—we find ourselves in similar states of mind and body. This is the remarkable fact about living systems. All other self-organising systems, from snowflakes to solar systems, follow an inevitable and irreversible path to disorder. Conversely, biological systems are characterised by a random dynamical attractor—a set of attracting states that are frequently revisited. Indeed, the characteristics by which we define living systems are simply statements about the characteristic, attracting states in which we find them [32]. This set of attracting states can be interpreted as the extended phenotype of the organism—its morphology, physiology, behavioural patterns, cultural patterns, and designer environments [34]. This conception of the extended phenotype as the set of attracting states of a coupled dynamical system is supported by evidence from simulation studies of morphogenesis, e.g., [28]. Further supportive evidence comes from studies of cancer genesis and progression, where the success of approaches employing endogenous networks provides a striking example of employing statistical methods (the Markov blanket formalism) to separate internal (phenotypical) states from external ones [35]. This conception of the topology of the phase space is supported by recent work on early myelopoiesis in real biological systems as well [36]. In this study, the core molecular endogenous network under consideration was cast as a set of dynamical equations, yielding structurally robust states that can be interpreted in relation to known cellular phenotypes.

The implications of this are profound. It means that all biotic agents move, systematically, towards attracting states (i.e., those with high probability) to counter the dispersive effects of random fluctuations. Consequently, any living system will appear, on average, to move up the probability gradients that define its attracting set—and the very characteristics responsible for its existence. Thus, living systems do not just destroy energy gradients (by gravitating towards free energy minima), they also create and maintain them by climbing the probability gradients that surround such extrema. In other words, living systems carve out and inhabit minima in free energy landscapes, precluding the dissipation of their states over phase space. This (nonequilibrium steady-state) behaviour differentiates living states from other states, like decay and death [1], [37], [38], [39]. Technically, this gradient-building behaviour can be expressed as the flow over a landscape that corresponds to the log probability of any state being occupied. This probability is also known as ‘Bayesian model evidence’ [26]. This means living systems are effectively self-evidencing—they move to maximise the evidence of their existence [40]. So how do they achieve this?

**Fig. 1:**
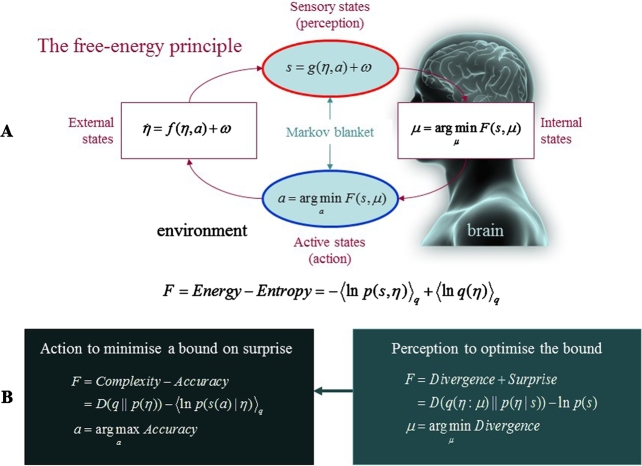
The free energy principle. (**A**) Schematic of the quantities that define free-energy. These include the internal states of a system *μ* (e.g. a brain) and quantities describing exchange with the world; namely, sensory input *s* = *g*(*η*,*a*)+*ω* and action *a* that changes the way the environment is sampled. The environment is described by equations of motion, $\overset{˙}{\eta }=f(\eta ,a)+\omega$, that specify the dynamics of (hidden) states of the world *η*. Here, *ω* denote random fluctuations. Internal states and action both change to minimise free-energy, which is a function of sensory input and a probabilistic representation (variational density) *q*(*η*:*μ*) encoded by the internal states. (**B**): Alternative expressions for the free-energy illustrating what its minimisation entails. For action, free-energy can only be suppressed by increasing the accuracy of sensory data (i.e., selectively sampling data that are predicted). Conversely, optimising internal states make the representation an approximate conditional density on the causes of sensory input (by minimising divergence). This optimisation makes the free-energy bound on surprise tighter and enables action to avoid surprising sensations.

This is where the FEP comes in. It asserts that all biological systems maintain their integrity by actively reducing the disorder or dispersion (i.e., entropy) of their sensory and physiological states by minimising their variational free energy ([26]; see Fig. 1 and Box 2). Because the repertoire of functional or adaptive states occupied by an organism is limited, the probability distribution over these characteristic states has low entropy: there is a high probability the organism will revisit a small number of states. Thus, an organism's distal imperative of survival and maintaining functional states within physiological bounds (i.e., homeostasis and allostasis) translates into a proximal avoidance of surprise [26]. Although surprise itself cannot be evaluated, since free energy imposes an upper bound on surprise, biological systems can minimise surprise by minimising their variational free energy. From the point of view of a physicist, surprise corresponds to thermodynamic potential energy [41], such that minimising (the average) variational free energy entails the minimisation of thermodynamic entropy. Consistent with EST, this propensity to minimise surprise is the result of natural selection (that itself can be seen as a free energy minimising process; see below)—self-organising systems that are able to avoid entropic, internal phase-transitions have been selected over those that could not [25]. This begs the question of how biological systems minimise free energy. To answer this, we will now take a closer look at the relation between surprise and (variational) free energy by introducing the notion of Markov blankets, which is central to the variational neuroethology described later.

### 2.2 Free energy, surprise, and Markov blankets

To understand the subtle but important difference between surprise and free energy, we have to look more carefully at what constitutes a system. Clearly, one needs to differentiate between the system and its environment—those states that constitute or are intrinsic to the system and those that are not. To do this, we have to introduce a third set of states that separates internal from external states. This is known as a Markov blanket. Markov blankets establish a conditional independence between internal and external states that renders the inside open to the outside, but only in a conditional sense (i.e., the internal states only ‘see’ the external states through the ‘veil’ of the Markov blanket; [32], [42]). The Markov blanket can be further divided into ‘sensory’ and ‘active’ states that are distinguished in the following way: internal states cannot influence sensory states, while external states cannot influence active states [43]. With these conditional independencies in place, we now have a well-defined (statistical) separation between the internal and external states of any system. A Markov blanket can be thought of as the surface of a cell, the states of our sensory epithelia, or carefully chosen nodes of the World Wide Web surrounding a particular province.

With Markov blankets in mind, it is fairly straightforward to show that the internal states must—by virtue of minimising surprise—encode a probability distribution over the external states; namely, the causes of sensory impressions on the Markov blanket. This brings us back to free energy. Free energy is a functional (i.e., the function of a function) that describes the probability distribution encoded by the internal states of the Markov blanket. Note that this is different from surprise, which is a function of the states of the Markov blanket itself. In other words, free energy is a function of probabilistic beliefs, encoded by internal states about external states (i.e., expectations about the probable causes of sensory input). When these beliefs are equal to the posterior probability over external states, free energy becomes equivalent to surprise. Otherwise, it is always slightly greater than (i.e., imposes an upper bound on) surprise. This means that living systems can be characterised as minimising variational free energy, and therefore surprise, where the minimisation of variational free energy entails the optimisation of beliefs about things beyond or behind the Markov blanket (see Box 2). This inferential aspect leads us to notions like embodied inference [44], [45], the Bayesian brain [46], the Bayesian cell [27], and even a Bayesian culture [29], [47].

In short, then, how do Markov blankets relate to the FEP? The FEP tells us how the quantities that define Markov blankets change as the system moves towards its variational free energy minimum (following Hamilton's principle of least action; see Fig. 1 and Box 2). It asserts that for a system to resist entropic erosion and maintain itself in a bounded set of states (i.e., to possess a generalised homeostasis), it must instantiate a causal, statistical model of its eco-niche relation [26], [32], [48], [49]. In other words, an organism does not just encode a model of the world, it *is* a model of the world—a physical transcription of causal regularities in its eco-niche that has been sculpted by reciprocal interactions between self-organisation and selection over time. On the basis of these distinctions, we turn next to defining a fully generalizable ontology for biological systems based on a multiscale free energy formulation, which we call ‘variational neuroethology’.

## 3 The big picture: a multiscale free energy formulation

The crux of our argument is that organisms can be described in terms of a (high dimensional) phase space induced by hierarchically nested Markov blankets. In other words, our ontology comprises populations of both spatially and temporally nested Markov blankets that occupy hierarchically nested regions in the total phase space of living systems. This sort of hierarchical organisation is a direct corollary of EST: since specific, functional (global) patterns of interacting (local) components need to be selected over competing alternatives to allow different levels of (informational, physical, chemical, biological, psychological, and sociocultural) organisation to emerge, the hierarchical nesting of Markov blankets instantiates Darwinian dynamics, which follows the same laws of statistical (or, strictly speaking, stochastic) thermodynamics, but in a nonequilibrium context that leads to self-organisation, self-assembly, and selective dynamics [7], [31], [32], [50].

### 3.1 Nested Markov blankets

**Fig. 2:**
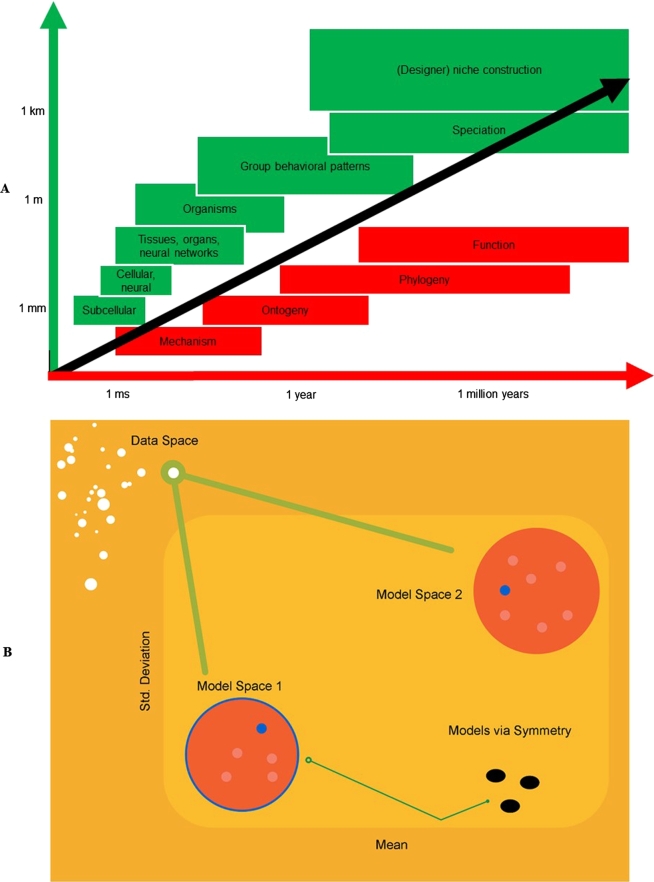
Variational neuroethology. (**A**) The meta-theoretical ontology we propose, called ‘variational neuroethology’, uses the FEP to explain and predict how living systems instantiate adaptive free energy minimisation. We have indicated some scales at which free energy minimizing dynamics unfold. Since spatial and temporal scales are intrinsically correlated (i.e., events unfolding over long distances usually take more time to unfold), what we have is a scale space that is populated mostly along its diagonal [55], [56], [57]. (**B**) Equivalence classes of variational free energy minimizing systems. The free energy minimising dynamics at play are implemented by different kinds of mechanisms in different individual organisms and species, as a function of the coupling between their evolved phenotypes and biobehavioural patterns and the niches they inhabit and the scales under scrutiny. The gauge theoretical formalism for the FEP [65] allows us to computationally model the regions of the biotic phase space, along its diagonal, that are apt to realize equivalent classes of dynamics. From [65].

To picture such hierarchical dynamics, it is useful to introduce the notion of a scale space. Scale spaces allow us to observe structures at different spatial scales. Imagine that you took a photograph, and then focused in progressively to examine smaller details. As you zoom in, you traverse a (spatial) scale space. The notion of a scale space is useful because the increase in scale, as we move from one hierarchical level of Markov blankets to the next, necessarily entails an increase in spatial scale. However, what were purposeful (i.e., free energy minimising) fluctuations at one scale now become fast random fluctuations at the next. This means that there is a concomitant increase in temporal scale as we ascend the spatial hierarchy. This composition of temporal and spatial scales is evident in the hierarchical organisation of the brain [51], [52], and more broadly, suggests that self-organisation should occupy a limited domain (along the diagonal) of a scale space with spatial and temporal dimensions [18] (see Fig. 2). Note that the use of a scale space is purely for descriptive purposes. The underlying system in question does not change—just its level of description, the way it is measured, or the perspective taken on its hierarchical self-organisation.

**Fig. 3:**
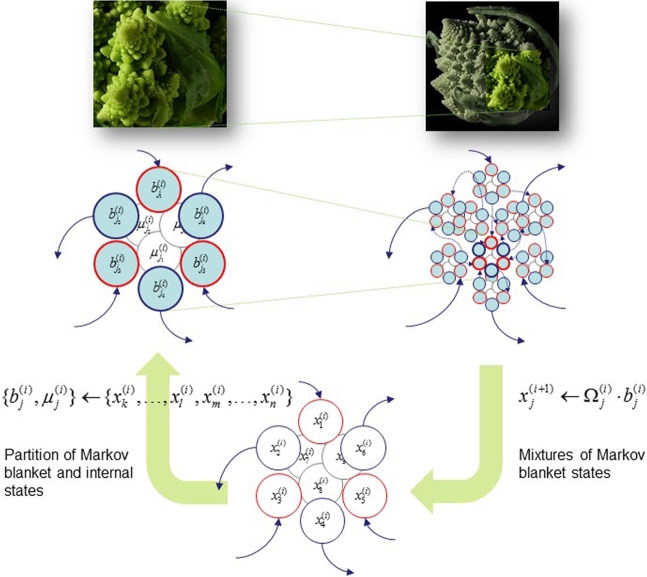
Nested Markov blankets. This schematic illustrates the hierarchical construction of (scale-free) compositions of Markov blankets of Markov blankets. The idea here is that particles, cells or subsystems at one scale (each comprising a Markov blanket $b_{j}^{\left(i\right)}$ that enshrouds internal states $\mu_{j}^{\left(i\right)}$) constitute an ensemble of states with a sparse dependency structure, which induces a Markov blanket at the supraordinate scale. This allows one to construct Markov blankets of Markov blankets by (i) partitioning the states at one level into a series of internal subsets and their Markov blankets and (ii) creating states for the next level by taking mixtures of Markov blanket states. Note that the internal states can be ignored when going from one level to the next because they are conditionally independent of external states (i.e., mixtures of Markov blankets from other subsystems). The mixtures can be regarded as slow (unstable) modes that are referred to as *order parameters* in synergetics [18]. Filled (cyan) circles correspond to Markov blanket states at the i-th scale, where, as in Fig. 1, red denote sensory states and blue active states. The pictures (of Broccoli) in the upper panels illustrate the self similarity of this recursive partitioning. (For interpretation of the references to colour in this figure, the reader is referred to the web version of this article.)

Recall from above that every ergodic system must possess an (ergodic) Markov blanket [32]. This simple observation delivers us to the core of our argument. Thus, we should be able to describe the universe in terms of Markov blankets of Markov blankets—and Markov blankets all the way up, and all the way down (see Fig. 3 and Box 3). To unpack this, consider an ensemble of cells, each equipped with a Markov blanket that corresponds to the cell surface. Because the internal states of each cell are sequestered behind their respective Markov blankets, all interactions between cells must be mediated by their Markov blankets. This means that we can describe the self-organisation of the cellular ensemble purely in terms of transactions among the (sensory and active) states of Markov blankets. However, these exchanges will themselves have a sparsity structure that induces a Markov blanket of Markov blankets. For example, one subset of the ensemble could be an organ that is surrounded by epithelia that provide a Markov blanket for all of the cells that constitute the internal states of the organ. However, this still follows exactly the same (statistical) structure—an ensemble of Markov blankets. We can then repeat this process, at increasingly larger scales of self-organisation, to create a series of hierarchically nested systems (e.g., the body) [42].

This sort of hierarchical structure provides a universal and recursive perspective to understand self-organisation across spatial and temporal scales, and to explain how each level contextualises (constrains) the levels both above and below. The hierarchal composition of Markov blankets within Markov blankets follows naturally from the existence of a Markov blanket that, in turn, is mandated by the existence of any system that can be distinguished from its external milieu. The key point here is that at every level, the same variational, surprise-reducing dynamics must be in play to supply Markov blankets for the level above. As we argue below, this idea offers a promising new research heuristic for the biological sciences.

### 3.2 Multiscale integration and variational neuroethology

Above, we suggested that living systems can be described in terms of hierarchically nested Markov blankets. The hierarchical interdependencies between Markov blankets provide a context for biological phenomena at every scale, which implies that the preservation of Markov blankets at a lower scale are necessary for the ongoing conditional independencies that form the basis of Markov blankets at a higher scale, and vice-versa (see Fig. 3 and Box 3). Put simply, the existence of every level depends upon every other level. Of particular interest here, however, is the importance of the different temporal scales that transcend spatial scales. Strictly speaking, free energy is only ever minimised diachronically—that is, over some discrete time span—as a process [53]. After all, the FEP is a formulation of the constraints imposed on system dynamics: it tells us about the dynamics of living systems that obtain precisely because of what it means for physical systems to be alive [43].

The hierarchical free energy formulation we propose here encompasses the ways in which the FEP applies to each hierarchical level of organisation by articulating the minimisation of free energy across scales. Any system's operation or functioning (i.e., a particular, time extensive event in our ontology) can be seen as unfolding across spatial scales, driven by temporally integrated free energy minimisation dynamics. Because the time average of free energy is a Hamiltonian action, all we are saying here is that Hamilton's principle of least action (perhaps the most celebrated variational principle) is realised by hierarchically nested biophysical processes. Having said this, the assertion that the time average of free energy is a Hamiltonian action is non-trivial for general stochastic dynamics, and is still a subject of research, i.e., [54]. Over any given time lapse, free energy minimisation can thus be framed as a hierarchical ‘unpacking’, across spatial scales, of the same invariant (or scale-free) dynamics. Since spatial and temporal scales are intrinsically correlated (i.e., events unfolding over long distances usually take more time to unfold [55], [56], [57], what we have is a scale space that is populated mostly along its diagonal (see Fig. 2).

Framing things in this way allows us to derive a computationally tractable, dynamical typology of the scientifically relevant events (types of biotic systems) at each timescale, and also to distinguish between different regions in the biophysical phase space, which roughly correspond with the ontologies of scientific disciplines concerned with different living systems (e.g., biology, psychology and the social sciences; [58]). These self-organising, variational imperatives remain identical at each level of organisation, but differ fundamentally in how they are mediated. For example, the brain uses some form of belief propagation or predictive coding to maintain its Markov blanket [59], [60], [61], while evolution uses Bayesian model selection and stochastic sampling schemes [15], [62], [63], [64] to preserve the Markov blankets that underlie speciation at evolutionary timescales.

The notion of Markov blankets allows us to define a formal ontology of living systems. Markov blankets are nested within Markov blankets, over spatial and temporal scales. This suggests that there has to be an internal (scale-free or scale-invariant) consistency, in the sense that the variational free energy associated with a global Markov blanket (e.g., a species) has to conform to the same principles as all of its constituent Markov blankets (e.g., phenotypes), which applies recursively right down to the level of biological macromolecules. In this vein, the FEP has recently been formulated using the resources of gauge theory [65]; see also the treatment of dynamic systems in [66]. Gauge theory is a family of mathematical models that have been broadly applied in physics. The gauge theoretical formalism is used to define a Lagrangian, a functional that summarises the dynamics of a given system and preserves its global symmetry (see Box 4). This symmetry is broken by local forces, invoking a gauge field that restores the system's symmetry by compensating for these local perturbations. The FEP has been proposed as the Lagrangian of a gauge theory for living systems [65]. Over any given time scale, free energy is minimised across every spatial scale, while local dynamics at each of these scales—determined by local Markov blanket features—perturb the Lagrangian. However, these perturbations are then compensated for by one or more gauge fields, which are introduced when an organism either changes its internal (e.g., perceptual) states or acts upon the world to minimise surprise. The upshot of this is that it allows us to computationally model scale-invariant free energy minimisation dynamics across temporal and spatial scales. As we ascend nested Markov blankets, and as we consider different biological systems, free energy is minimised in dynamically equivalent, but mechanistically heterogeneous, ways.

This brings us to a meta-theoretical ontology of biological systems derived from the FEP—variational neuroethology—that can be used to explain and predict how living systems, at any spatial and temporal scale, instantiate the dynamics of adaptive free energy minimisation. The precise nature of free energy minimising mechanisms will vary from organism to organism and species to species, as a function of their evolved phenotypes and biobehavioural patterns. In other words, the FEP supplies a universal Lagrangian that extends across all spatial and temporal scales, but the particular ways in which it is implemented will vary according to the species, organisms, and scales under scrutiny. This allows us to computationally model the regions of the phase space populated by different organisms—along its diagonal—that realise equivalent classes of dynamics, and to recast the issue of ecological problem-solving as the problem of finding local free energy minima. At first glance, analysing such complex dynamics across time and space might seem intractable, but conveniently, biologists have long been familiar with a highly compatible framework that allows us to translate our variational neuroethology into viable scientific practice.

## 4 Integrating the multiscale free energy formulation with Tinbergen's four questions

In the sciences of life, mind, and society, using free energy minimisation—realised by nested Markov blankets—enables us to explain dynamics both at and between nested scale spaces. However, although the FEP supplies a powerful, mechanistic theory that captures biological dynamics across spatial and temporal scales, it can only offer partial insight into the particular features of a given species, which instantiate distinct, embodied models of specific adaptive needs and environmental niches [34], [67], [68]. In his treatment of Darwinian dynamics, Ao [5] describes how a complete explanation for any biological system requires two types of laws: those that account for the structure of evolutionary dynamics (e.g., natural selection); and those that can explain the structure of each dynamical component (e.g., genes). We suggest here that the FEP is analogous to the first type of law—it describes, formally, the (entropy bounding) dynamics of all living systems. But what of the second type? Although it is highly generalizable, the explanatory scope of the FEP is limited—it only imposes relatively modest constraints on the classes of dynamical patterns (i.e., complex adaptive systems) that count as living. The FEP therefore requires a complementary evolutionary (i.e., ultimate) account that explains the specific adaptive solutions responsible for producing different embodied models, along with the proximate processes that produce every phenotype. Again, this appeals to the importance of timescales when describing the dynamic ways in which living systems minimise free energy across space and time.

Fortunately, the importance of temporal scales has long been recognised in biology, particularly after Tinbergen [69] proposed his four key levels of biological explanation (i.e., adaptation, phylogeny, ontogeny, and mechanism). Given the success of this explanatory framework in biology, we suggest that Tinbergen's levels of inquiry might be apt to elucidate structural laws that supplement the general principles provided by the FEP. Clearly, Tinbergen's ‘four questions’ have a long history of producing detailed explanations for the evolution, development, form and function of different species, and by focusing on different temporal scales (ranging from a species' evolution to an organism's behaviour in real-time), they allow us to capture the complexities of every corresponding spatial scale (from sub-atomic particles, atoms, and cells, all the way up). Conversely, the FEP furnishes a biologically plausible EST that applies both to ultimate and proximate processes, supplying a universal principle that intersects all four of Tinbergen's levels of inquiry. We believe, then, that these two paradigms are highly commensurate—the FEP describes a biological imperative to model the world that constrains the dynamics of all living systems (at any time-scale), while Tinbergen has offered a distinctive but complementary framework that allows us to develop substantive explanations for the phenotypic traits and behaviours of any given species or organism. Accordingly, the variational neuroethology proposed here hinges on their synthesis. To exemplify this approach, we turn now to its recent application to the world's most complex living system—us.

### 4.1 Variational neuroethology applied: hierarchically mechanistic minds

The FEP is best known in neuroscience, where it has been used to explain the structure, function, and dynamics of the brain. In this context, the FEP concords with predictive coding by describing the brain as a hierarchical ‘inference machine’ that minimises prediction error by seeking to match incoming sensory inputs with top-down (neuronally-encoded) predictions [26], [34], [40], [68]. This occurs in two ways: we can either improve our predictions by altering internal states (i.e., perception); or we can act upon the world to confirm our predictions (i.e., action). Thus, action and perception operate synergistically to optimise an organism's (Bayesian) model of the environment. As discussed, the FEP also transcends predictive coding by extending beyond the brain to explain behaviour, the phenotype, and all other biotic phenomena that span evolutionary timescales and spatially distributed ensembles. However, to understand the particular features of the human brain, the FEP requires recourse to research in psychology and the social sciences (e.g., evolutionary and cognitive anthropology), which target the specific ecobiopsychosocial processes responsible for the embodied models and behaviour of Homo sapiens in particular.

**Fig. 4:**
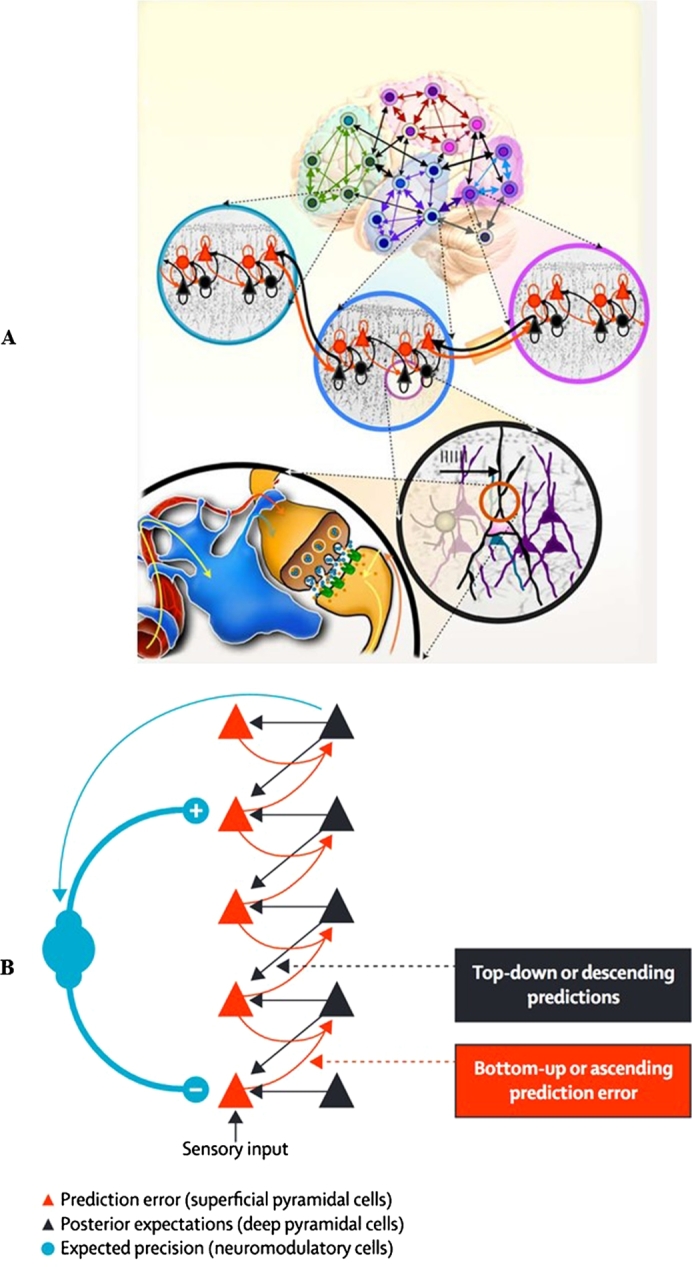

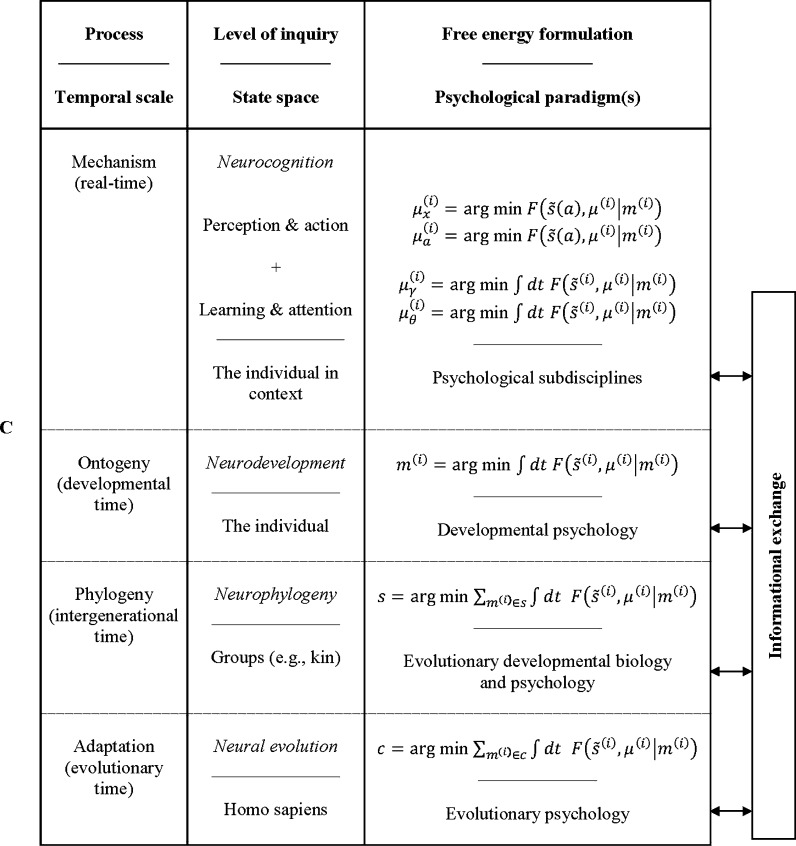
The hierarchically mechanistic mind. (**A**) Schematic of the multiscale hierarchical organisation of brain networks. Neural networks are composed of nodes and their connections, called edges. A node, defined as an interacting unit of a network, is itself a network composed of smaller nodes interacting at a lower hierarchical level, producing nested neural networks that extend from neurons, to macrocolumns, and to macroscopic brain regions. From [72]. (**B**) A simple cortical hierarchy with ascending prediction errors and descending predictions. Superficial pyramidal cells (red triangles) compare expectations (at each level) with top-down predictions from deep pyramidal cells (black triangles) at higher levels. Neuromodulatory gating or gain control (blue) of superficial pyramidal cells determines their relative influence on the deep pyramidal cells that encode expectations by modulating their precision. From [93]. (**C**): The variational neuroethology of human cognition and biobehaviour. $F(\overset{˜}{s}(a),\mu^{\left(i\right)}|m^{\left(i\right)})$ represents the free-energy of the sensory data (over time), $\overset{˜}{s}(a)$, and the states *μ* of an agent *m*^(*i*)^ ∈ *s* that belongs to a subgroup *s* ∈ *c* of class *c*. Action (*a*) governs the sampling of sensory data, and the physical states of the phenotype (*μ*) encode beliefs or expectations (and expectations of the mean of a probability distribution). Free energy minimisation dynamics vary across timescales, ranging from neurocognition in real-time (i.e., perception and action; learning and attention); neurodevelopment throughout the lifespan; epigenetic mechanisms that minimise free energy across generations (e.g., kin); and the process of adaptation, which involves the optimisation of human generative models over time and conspecifics via the inheritance of adaptive priors [104]. These temporal processes are captured by Tinbergen's four levels of inquiry, which appeal to a dynamic causal hierarchy that is encapsulated by complementary paradigms in psychology: evolutionary psychology; evolutionary developmental approaches; developmental psychology; and the psychological subdisciplines [7], [70]. (For interpretation of the references to colour in this figure, the reader is referred to the web version of this article.)

To address this need, an interdisciplinary EST of the embodied brain has recently been forwarded called the hierarchically mechanistic mind (HMM). Initially proposed to unify evolutionary and developmental psychology, the HMM is an evidence-based model of neurocognition and biobehaviour that synthesises the FEP with major paradigms in psychology to situate the brain within the broader evolutionary, developmental, and real-time processes that produce human behaviour, phenotypes, and niches [7], [70]. Specifically, this model defines the human brain as an embodied, complex adaptive system that adaptively minimises the entropy of its internal (i.e., sensory and physiological) states through recursive interactions between hierarchically organized, functionally differentiated neural subsystems [7]. This hierarchy ranges from lower-order, highly segregated neurocognitive systems responsible for sensorimotor processing, through to the highly integrated association areas that underlie the sophisticated cognitive faculties unique to humans [70]. The HMM resonates with structural and functional imaging studies in network neuroscience, which show that the brain entails a multiscale hierarchical organisation characterised by the repeated encapsulation of smaller neural elements in larger ones ([71], [72]; Fig. 4, panel A). Predictive coding approaches suggest that this sort of architecture entails a hierarchical generative model that minimises prediction error via recurrent message-passing between cortical levels (Fig. 4, panel B), affording a neurobiologically plausible, mechanistic theory of the functional integration of anatomically segregated neural networks [72], [73], [74].

Following EST, the hierarchical brain also exemplifies the complementary relationship between evolution and development—selection has canalized early sensorimotor regions that serve as neurodevelopmental anchors, allowing for the protracted, activity-dependent self-organisation of ‘domain-general’ association cortices throughout development that enhance evolvability by responding flexibly to environmental change [75], [76]. Consistent with this, simulation studies of evolving networks have shown that a hierarchical neural structure enhances evolvability by adapting faster to new environments than non-hierarchical structures [77]. The hierarchical segregation of neural networks into distributed neighbourhoods has also been found to stretch the parameter range for self-organized criticality by allowing subcritical and supercritical dynamics to co-exist simultaneously [78], which optimizes information processing and is therefore likely to be favoured by selection [79]. Maturational studies of neural networks throughout childhood and adolescence have further revealed that human cortical development mirrors phylogeny, progressing from the sensorimotor hierarchies found in all mammals through to recent association areas shared by humans and other primates [80]. The phylogeny of the brain is also reflected across nested levels of neurophysiological organisation, ranging from genes and transcription factors through to synaptic epigenesis and the long-range connectivity that is thought to underpin consciousness [81]. It is unsurprising, then, that a hierarchical neural structure has been found in every mammal examined to date [82].

The HMM rests on two assumptions. First, it subsumes the FEP as a biologically plausible EST of the evolution, development, form, and function of the brain. The FEP suggests that instead of just encoding a model of the world, the brain itself manifests a (hierarchical) phenotypic transcription of causal structure in its environment (i.e., an embodied statistical or generative model) that is optimized by evolution, development, and learning. Here, selection is simply nature's way of performing Bayesian model optimisation by minimising the (variational) free energy of human phenotypes across different (Tinbergian) timescales (Fig. 4, panel C). Emphasis is placed on adaptive priors, which are (epi)genetically-specified expectations that have been shaped by selection to guide action-perception cycles toward adaptive or unsurprising states [26].

The second assumption follows EST in psychology by recognising that the brain is an emergent sub-system produced by a hierarchy of causal mechanisms that act on the brain-body-environment system over time (i.e., adaptation, phylogeny, ontogeny, and mechanism; [7], [53], [83]). This causal hierarchy is recapitulated by major paradigms in psychology—which focus differentially on Tinbergen's [69] four questions (Fig. 4, panel C)—and calls for sophisticated, multiscale hypotheses that synthesise the FEP with different research programs in psychology and anthropology to explain both why a particular trait is adaptive, along with how it emerges from intergenerational, developmental, and real-time processes [7], [70]. The FEP and EST in psychology can therefore be regarded as different sides of the same coin—the FEP supplies a fully generalizable mechanistic theory of neural structure and function that applies to species generally, while an evolutionary systems approach to psychology builds upon the FEP by providing a substantive, evolutionary framework that is capable of explaining how the FEP manifests in Homo sapiens [84].

### 4.2 Translating variational neuroethology into research heuristics

The HMM is not just a second-order theory derived from variational neuroethology; it can also be translated into a multidisciplinary research heuristic that promotes a tripartite approach to scientific inquiry [70]. First, it calls for integrative, multilevel hypotheses in psychology that address all four of Tinbergen's questions by synthesising research across evolutionary psychology, biological and cognitive anthropology; evolutionary developmental biology and psychology; developmental psychology; and psychological sub-disciplines such as cognitive, social, biological and personality psychology (Fig. 4, panel C). Second, it requires an analysis of how the phenomenon of interest can be explained in terms of the FEP. Finally, it calls for empirical research into the ways in which the phenomenon is neurophysiologically instantiated in a dynamic, hierarchical manner and manifests behaviourally via active inference [85]. Although this synergistic approach to developing hypotheses is undoubtedly complex and remains in its infancy, it has already furnished important new insights into depression [70], and yields distinct implications for neuroscientists and psychologists alike, while creating new avenues for collaborative research. This variational neuroethology of human systems has the major methodological advantage of conferring bona fide predictive power to the biological, cognitive, and social sciences. We can use simulation studies to predict which solutions will be adopted to respond adaptively to a given ecological problem, and then compare these computational models with empirical data.

For neuroscientists, the HMM lends itself to multimethod approaches that explore how detectable regularities in measurements of the brain can be explained by the psychological factors responsible for different patterns of neural activity in different contexts [86]. One way to isolate these factors is to use large databases of task-based fMRI activation studies to characterize the functional fingerprints of specific neural regions across different task demands [86], while another is to use such datasets or combine functional activation studies with neuropsychological lesion-deficit models to derive cognitive ontologies that systematically map relationships between specific cognitive functions and hierarchical neural dynamics [87], [88]. More broadly, the HMM also encourages the uptake of traditional methods in psychology—such as observational data collection and interviews—to explore the intersections between mind, brain, and behaviour [70]. Approaches in developmental psychology can be used to explore the dynamic ways in which human development differentiates (error-minimising) neurocognitive and biobehavioural patterns between individuals [70], while comparative, cross-cultural, computational and dynamical approaches in evolutionary psychology and cognitive anthropology can elucidate the (epi)genetic mechanisms that underlie our species-typical adaptive priors [29], [84], [89], [90]. Finally, dynamical methods such as computer simulations and computational models enable us to directly examine how such levels of causation interact [91], [92], [93], allowing neuroscientists to discover how the phenomena highlighted by psychologists reflect adaptive free-energy minimisation under different (evolutionary, developmental and ecobiopsychosocial) conditions. The outcomes of such analyses might then be confirmed through experimental research, potentiating a fruitful dialectic between computational analyses and real-world observations.

On the other hand, the FEP offers a neurobiologically plausible EST of the mind and biobehaviour to psychologists. It has already been fruitfully applied to a wide range of psychological phenomena [68], [94], extending from emotion (e.g., [95]), anxiety (e.g., [96]) and depression (e.g., [70]), through to autism (e.g., [97]), illusions (e.g., [98]) and psychosis (e.g., [99]). The FEP also lends itself to methods that are highly familiar to psychologists, such as the P300; a psychophysiological measure that captures temporal fluctuations in surprise [100]. Finally, since it can be equally applied across all four of Tinbergen's research questions, the FEP casts new light on the reciprocal interplay of ultimate and proximate processes; stands to benefit both evolutionary and developmental psychologists; and proffers a common, transdisciplinary language to unite psychology's sub-disciplines [7], [70].

However, if human phenotypes can only be understood by analysing the ecology from which they emerge, how might the HMM incorporate sociocultural influences—arguably our most influential selection pressure to date [89], [90], [101]? A promising way to address this question is to incorporate recent work on cultural affordances. According to this perspective, cultural ensembles minimise free energy by enculturing their members so that they share common sets of precision-weighting priors [29]. Human beings—with our specific forms of neural organisation, phenotypes, evolved behavioural tendencies and sociocultural patterns—minimise more free energy across spatial and temporal scales than any other species. Arguably, this is because we have crossed the ‘evolutionary Rubicon’: our survival depends on our ability to access and leverage cultural information and immersively participate in culturally adapted practices [89]. This is just another coordinated set of nested spatial and temporal scales in the greater Markov blanket of Homo sapiens. Another scale to consider—which we share with other animals like beavers and bees—is the free energy minimisation accomplished by designer environments [68]. Although the ways in which the HMM might be extended to incorporate cultural affordances remains an open question, a promising avenue would be to explore approaches in scientometrics. Using science as the subject of its inquiry, this discipline incorporates a wealth of models and quantitative techniques (e.g., citation and text analyses) that can be used to analyse how general selection and self-organisation act upon theorizing and research to reduce uncertainty about the environment [102], [103]. This affords a promising means to explore how the sociocultural generative models instantiated by different disciplines optimise (scientific) model selection by minimising free-energy (i.e., scientific prediction errors) over time [7], [34].

Ultimately, the HMM and theory of cultural affordances both rely on the idea that the Markov blanket of Homo sapiens possesses hierarchically nested temporal and spatial dimensions. These models both appeal to a scientific meta-theory (i.e., variational neuroethology) that defines Homo sapiens as each individual (phenotype) throughout the course of evolution, along with every individual that has either existed in the past or occupies the present (i.e., our species)—who may, in turn, alter the course of our evolution and our characteristically adapted niches. This is just another way of saying that the Markov blanket constituted by a single phenotype is dynamically nested within the global Markov blanket of our species (extending across all of Tinbergen's temporal scales). Conveniently, this approach also allows researchers to navigate the recursive complexities of the spatial axis (from biological macromolecules all the way up). By placing our Markov blanket around Homo sapiens, we necessarily encapsulate all of the dynamic, lower-level processes responsible for producing every phenotype, while imposing a clear upper limit on the complex adaptive system under scrutiny. Although the human Markov blanket is nested within the broader dynamics of other global Markov blankets that extend out into the universe, these lie beyond the limits of the system that this ecobiopsychosocial framework endeavours to explain.

## 5 Concluding remarks

The purpose of this article was to answer Schrödinger's question – ‘what is life?’ – by presenting a hierarchical multiscale free energy formulation—called variational neuroethology—that offers the sciences of life, mind, behaviour and society with a principled, computationally tractable guide to discovery. Arguably, the FEP affords a unifying perspective that explains the dynamics of living systems across spatial and temporal scales. The ontology for biological systems discussed here entails a multidimensional phase space populated by events (i.e., living systems as adaptive, self-organising patterns) that depend on the temporal dynamics of free energy minimisation. These events can be described as spatially and temporally nested Markov blankets, the dynamics of which are summarised by the Lagrangian of the FEP. Synthesising this framework with Tinbergen's four questions allows us to frame our scientific investigations with clearly defined temporal and spatial bounds, depending on the specific questions we wish to answer. We have described an empirically supported exemplar of this approach in the cognitive and behavioural sciences—the HMM—and shown how this theory yields unique and promising avenues for interdisciplinary research. The challenge, of course, lies in translating theory into productive scientific practice, and in testing the current limits of the FEP by applying it to species without a brain, like E. coli, fungi and flora.

## Appendix A Supplementary material

Box 1 Dynamical systems theory. Box 1

Box 2 Variational inference and the free energy formulation. Box 2

Box 3 Nested Markov blankets. Box 3

Box 4 A gauge-theoretical free energy formulation and variational neuroethology. Box 4
