# Supplementary material for: Answering Schrödinger's question: A free-energy formulation
Source: Phys Life Rev. 2018 Mar;24:1–16. doi: 10.1016/j.plrev.2017.09.001 (PMC5857288; doi:10.1016/j.plrev.2017.09.001)
Supplement: Box 1 — Dynamical systems theory. [file mmc1.pdf]

Supplementary Materials:

Supplementary Information Box 1. Dynamical systems theory.

Evolutionary systems theory (EST), the ambient meta-theory that frames the free energy formulation, subsumes dynamical systems theory (DST). DST is a mathematical formulation of systems dynamics. Living systems have been productively modelled using the resources of DST, and so a few of the central concepts borrowed from DST by EST should be explicated. The core feature of dynamicist approaches is their emphasis on dynamics that unfold over time. The free energy formulation shares its dynamicist commitments with closely related approaches in cognitive neuroscience and psychology: the ecological and enactive approaches to cognition and behaviour. Indeed, the free energy formulation has been proposed as a computationally-tractable, mathematical formulation of enactivism from first principles [32, 37, 53, 67, 83]. For example, all the process theories that follow from the FEP, from predictive coding through to the belief propagation in decision-making, rest explicitly on formulating neuronal dynamics as a gradient descent on variational free energy [105].

Dynamical systems are mathematical models that are used to represent physical systems with temporally extended dynamics. These dynamics are expressed as systems of (ordinary, stochastic or random) differential equations that describe trajectories or paths through phase space. They are defined over states of the system and their flow depends upon the current value of the system states. The analytic intractability of some of these systems of equations led to the development of qualitative methods to study dynamical systems. The strategy here is to qualitatively describe the dynamical evolution of a system by describing the abstract space of all of its possible states. A '*phase space*' is an abstract representation of all the possible states of a system. It is an *n*-dimensional (usually metric) space, where each dimension corresponds to a state variable of the system (e.g., position, velocity, etc.). A point in this *n*-dimensional phase space is an *n*-tuple that can be interpreted as assigning a value to each variable along each dimension. Because a point in the phase space of a dynamical system specifies a value for every variable of the system, any given point in this space represents a complete description of the system at a given instant.

How does this help us analyse the dynamics of a system? In dynamical systems theory, time is represented not as a separate dimension, but as a *dynamical trajectory* or sequence of states through the phase space. This trajectory is determined by the *topology* of the phase space, which translates the constraints to which the system is subject. That is, the topology of the phase space translates the system of differential equations that govern the dynamics of the system being studied, usually in terms of attracting sets, manifolds or orbits [106-109].

DST has especially been employed to understand *self-organisation*. Self-organisation is ubiquitous is nature: weather patterns, like rainstorms and lightning, emerge spontaneously; fluids crystallize to form structured lattices; bubbles arise in sea foam, and pop. This occurs because the spontaneous emergence of self-organized dynamics increases the efficiency of energy gradient dissipation and entropy production within that system (i.e., it increases its internal order). Self-organised dynamics emerge around an energy gradient, and optimise the flow of energy in the system about that energy gradient, until the system succumbs to decay [110]. DST provides qualitative and computationally tractable formalisms to model such self-organising dynamics.
