# Supplementary material for: Answering Schrödinger's question: A free-energy formulation
Source: Phys Life Rev. 2018 Mar;24:1–16. doi: 10.1016/j.plrev.2017.09.001 (PMC5857288; doi:10.1016/j.plrev.2017.09.001)
Supplement: Box 2 — Variational inference and the free energy formulation. [file mmc2.pdf]

Supplementary Information Box 2. Variational inference and the free energy formulation.
Organisms do not have access to the 'true' probabilistic contingencies that describe the entire organism-niche system, that is, the actual relations of dependencies between environmental states and states of the organism. After all, the biotic system itself is 'hidden', as it were, behind a Markov blanket, which endows it with statistical independence from random fluctuations and other influences from the 'outside'. However, it does have access to quantities that define the variational free energy, and it can leverage the gradients defined by the free energy landscape to resists entropic erosion, through the process of 'active inference' [111, 112]. Here, the organism's action-perception cycles can be seen as self-evidencing [40]; that is, as producing evidence that allows it to infer its own existence.

<u>Self-evidencing and variational inference</u>

Formally, we can model this behaviour using the *variational* methods that were first developed by Feynman[113], and are now widely used in statistical mechanics and machine learning [114-117]. In the context of the free energy formulation, the internal states of the organism (i.e., internal states of the Markov blanket) are formally described as encoding a 'variational' density, which comes to approximate the 'true' (posterior) probability density that is embodied by the organism-niche system, namely, through gradient descent on the free energy landscape. This warrants further explication.

Because living systems are inferentially secluded behind their Markov blanket, the causes of their sensory states are represented using surrogate or fictive variables, η, which represent the system's 'best guess' as to the cause of its input. As such, on the free energy formulation, the internal states of the organism encode or embody a 'variational density', which represents the organism's 'best guess' as to the causes of its sensations through cycles of free energy minimisation (a.k.a. active inference).

The ensuing formalism draws on approximate Bayesian inference (e.g., variational approaches in machine learning). When the computation of a posterior probability scheme becomes intractable in Bayesian inference (e.g., due to high dimensional and nonlinear generative models), a common strategy is to approximate the 'true' posterior density over the model parameters with a simpler variational density, whose sufficient statistics can be optimised easily. Usually, this involves something called a *mean field approximation* in which a high dimensional posterior density is approximated with a product of marginal densities. These marginal densities then minimise variational free energy by passing messages (sufficient statistics) to each other. When this variational message passing is cast as a gradient descent on variational free energy, we obtain a DST version of approximate (variational) inference.

In the present (general) setting, the variational density is defined as a probability density, q, that is encoded or parameterized by internal states $\mu(t)$ of the system of interest, which is itself bounded by a Markov blanket and subject to free energy minimising dynamics. Thus, the internal states of the system induce a variational density over external states $\eta(t)$. This is a consequence of the formulation of the free energy as *surprise* plus *divergence*. This means that when the variational free energy functional is minimised, the divergence disappears and the variational density becomes the posterior density. At the same time, the variational free energy approximates surprise or (negative) log Bayesian evidence.

<u>Active inference and variational free energy</u>

So, although the organism cannot access the true posterior density (or surprise), it can nonetheless evaluate the variational free energy, because this quantity is a function of two quantities which it can access: the variational density that it encodes (which is parameterised by its internal states) and the sensations or sensory states of the Markov blanket that are contingent upon action. This brings us to a crucial observation. The only way we can actually change surprise is by changing the sensory states through action. This means to minimise surprise through minimising variational free energy, we need to change our internal states to make the free energy a good proxy for surprise and then we need to act to change our sensory states to actually reduce surprise. If we do this for long enough, the expected surprise or entropy of our sensory exchanges will be minimised (i.e., model evidence will be maximised) and we will be locally ergodic and self-evidencing. This is ‘active inference’.

The generative models that define free energy are probabilistic models of the eco-niche relation, the dynamical relation that couples the organism to its niche. The generative model is usually expressed as the joint probability of sensory states, $s(t)$, and their causes, $\eta(t)$, in the (external) environment. This joint probability is usually expressed in terms of a likelihood $p(s(t)|\eta(t))$ and prior beliefs $p(\eta(t))$. With this formalism in place, one can say the variational density – that is parameterised by internal states – is *encoded* or *embodied* by the internal states of the system. Conversely, we can say the generative model is *entailed* by the existence of a system equipped with a Markov blanket. Equivalently, we can also say that the generative model is *enacted* by the entire organism-niche system—that is, the conditional dependencies described by the generative model are brought forth in a process that is accomplished by exchanges between the organism and econiche.

Ergodicity and active inference

By virtue of the existence of an attracting or characteristic set of states (the extended phenotype), the dynamics of the random dynamical system must be locally *ergodic*. Living systems must maintain their ergodicity in order to remain alive – and thereby engage in some form of *active inference*. On this view, ergodicity is not merely an assumption that underwrites any principle of self-organisation. It is a definition of the living, complex adaptive (biological) systems we want to characterise. In other words, any system that does not maintain its (local) ergodicity cannot, by definition, possess characteristics that can be measured. The ergodicity of systems that possess a random dynamical attractor allows us to associate the long-term average of self-information or surprisal with the entropy of the probability distribution of occupying different states. This means that a tendency to minimise surprisal (or, equivalently, to maximise model evidence) is also a tendency to minimise (internal) entropy and thereby resist the second law of thermodynamics. In the sense that entropy is formally equivalent to the time average of a log probability, it corresponds to a Hamiltonian action. This means that all we are saying is that living systems conform to Hamilton's principle of least action, where action is entropy. In fact, we are saying a little bit more than this, we are saying that living systems conform to Hamilton's principle of least action via active inference – and the implicit minimisation of variational free energy.
