# Supplementary material for: Answering Schrödinger's question: A free-energy formulation
Source: Phys Life Rev. 2018 Mar;24:1–16. doi: 10.1016/j.plrev.2017.09.001 (PMC5857288; doi:10.1016/j.plrev.2017.09.001)
Supplement: Box 3 — Nested Markov blankets. [file mmc3.pdf]

Supplementary Information Box 3. Nested Markov blankets.

The ontology that we propose for living systems relies on the notion of nested Markov blankets. Markov blankets are a statistical description of dependencies in coupled systems. A Markov blanket comprises a set of systems states that separate internal and external states of any given system. More specifically, the existence of a Markov blanket entails a conditional independence between internal and external states. In turn, the Markov blanket itself can be divided into sensory and active states. These are characterized by the following relations: internal states cannot influence sensory states, while external states cannot influence active states. With these conditional independencies in place, we now have a well-defined (statistical) separation between the internal and external states of any system. This is relevant for our purposes because it tells us about what does, and does not, constitute a system.

To be equipped with a Markov blanket means the internal states can exhibit a selective openness to the outside, but only in a conditional sense. In the terms of dynamic systems theory, Markov blankets allow for random dynamical systems that are open to the external (environmental) states yet retain their own (statistical) integrity [32]. They are open precisely to the extent that they exist far from thermodynamic equilibrium, and are permeable to fluctuations that originate from the environment. Open systems are open to energy and information exchange with their environment. However, internal systems are enclosed, because they are segregated (i.e., statistically independent) from external perturbations, and as such, are statistically insulated from environmental dynamics. These perturbations could lead to an altered topology (or attractor landscape) and an ensuing loss of integrity (phenotype, ergodicity). Clearly, then, for the living system to persist, its ergodicity needs to be maintained. This is evidenced by the persistence of the Markov blanket and its maintenance through active inference [85].

Now consider an ensemble of Markov blankets that exchange with each other. Recall that free energy is a functional of the beliefs entailed by internal states. However, the states of the Markov blanket will, in the ensemble, depend upon other Markov blankets. This means that the free energy minimum (when pooled over an ensemble of Markov blankets) necessitates a collective inference, in which no individual constituent of the ensemble surprises another. This coherence – perhaps mediated by generalised synchrony across Markov blankets – follows naturally from the free energy formalism (see [28], for an example based upon cellular interactions in morphogenesis). Put simply, if we all have a common agenda and each play our role, fulfilling each other's expectations, we can collectively minimise surprise and maintain our ensemble of Markov blanket – and implicitly a Markov blanket of Markov blankets. This provides the necessary milieu for each individual to thrive in a surprise-minimising, uncertainty-reducing sense; which, in turn, enables their brains to minimise free energy at the level of neurophysiology—all the way down to macromolecules and quantum physics.

This hierarchal composition of Markov blankets of Markov blankets follows naturally from the existence of a Markov blanket – the existence of Markov blankets around the system of interest is mandated by the existence of any system that can be distinguished from its external milieu. A crucial aspect of the hierarchical organisation of nested Markov blankets is that, at each level, the internal states are dropped from the game, as it were. In other words, the only states of interest—as we are lifted from one level to the next—are the Markov blankets that contain all of the necessary information for interactions at each level. Here, the dynamics of internal states are absorbed into the random fluctuations that are countered by gradient building, surprise minimising, self-evidencing flows. In other words, random fluctuations at one level are the

inferential dynamics of internal states at the level below. The idea put forward in this paper is that as we ascend hierarchical scales, the fast fluctuations of internal states that are necessary to preserve the integrity of Markov blankets become fast random fluctuations that are averaged away at the scale above.

The hierarchical interdependencies provide a context for phenomena at each scale, which implies that the preservation of ergodic Markov blankets, at a lower scale, are necessary for the ongoing conditional independencies that form the basis of ergodic Markov blankets at a higher scale, and vice-versa. Thus, every level depends upon every other level: the central claim is that at every level, the same variational, surprise-reducing dynamics must be in play to provide Markov blankets for the next level. We call on this central observation to frame important relationships in hierarchical selection and self-organisation.
