# Supplementary material for: Answering Schrödinger's question: A free-energy formulation
Source: Phys Life Rev. 2018 Mar;24:1–16. doi: 10.1016/j.plrev.2017.09.001 (PMC5857288; doi:10.1016/j.plrev.2017.09.001)
Supplement: Box 4 — A gauge-theoretical free energy formulation and variational neuroethology. [file mmc4.pdf]

Supplementary Information Box 4. A gauge-theoretical free energy formulation and variational neuroethology

The ‘intentionality’ or ‘aboutness’ of living systems—that is, the directedness of the organism towards a meaningful world of significance and valence—emerges as a natural consequence of embedded adaptive systems that satisfy the constraints of the free energy formulation. For a living thing to be intentional just means that it entails a generative model (that necessarily includes prior beliefs about the way it should behave). To entail a generative model means that the organism vicariously enacts or brings forth the conditions that define it as a dynamically coupled, complex adaptive system. Put simply: active systems are alive if, and only if, there active inference entails a generative model. This makes the generative model of central importance to the free energy formulation, since it defines the form of life that an organism is seen to enact.

However, our modelling strategies need not be constrained by generative models. The nested Markov blanket formalism allows us to define a formal ontology of living systems independent of specific generative models. How? Recall that Markov blankets can be nested within Markov blankets, over spatial and temporal scales. This suggests that there has to be an internal scale-free or scale-invariant consistency: the free energy associated with a global Markov blanket (e.g., a species) must conform to the same principles as all of its constituent Markov blankets (e.g., phenotypes). This consistency must apply recursively, all the way down to the level of biological macromolecules.

The free energy principle has recently been reformulated using the resources of gauge theory [65]. Gauge theory is a family of mathematical models that have been productively used in nearly all areas of physics, from electromagnetism to relativity and quantum mechanics. Gauge theories define a kind of dynamics, based on a Lagrangian, which preserves a symmetry or invariant. This symmetry is broken by local forces. In turn, symmetry breaking recruits a gauge field, which compensates for the local perturbations, restoring the system symmetry.

The free energy functional has been proposed as the Lagrangian of a gauge theory for neural and biological systems [65]. Crucially, this means that it is possible to mathematically model invariant free energy minimisation dynamics across temporal and spatial scales. The gauge theoretical Lagrangian, after all, is constructed to be scale invariant. This kind of modelling is less constrained by specific generative models, because it is interested in the resolution of local dynamics through the effects of gauge fields (which, here, is interpreted as free energy minimisation via active inference). Thus, over any period of time, the scale-invariant free energy Lagrangian is minimised across spatial scales, recruiting different local gauge dynamics as a function of the different local forces. At each scale, the local dynamics, determined by local Markov blanket features, perturb the Lagrangian. These perturbations are then compensated by gauge fields, which in the free energy formulation are achieved through active inference (perception, action, evolution, niche construction, and so forth).

This means that we can draw broad equivalence classes between different forms of free energy minimisation and thereby connect behavioural patterns that, while isomorphic from the standpoint of free energy minimisation, are mechanistically implemented or realised in different ways. Thus, the gauge-theoretical formulation allows us to define classes of equivalent internal states, patterns of action, and generative models (pitched at different temporal and spatial scales) that minimise free energy in systematically or dynamically equivalent, but mechanistically heterogeneous, physical systems. The gauge theoretical framework suggests that different classes

of generative models are able to guide the same (or analogous) dynamics, which points towards the possibility of developing a computational neuroethology based on the free energy formulation—which we have called ‘variational neuroethology’.

Living systems that conform to the principles described here will appear to minimise their free energy. However, this minimisation of free energy is not an additional force of nature or élan vital. Rather, it is a fictive force, which is ultimately due to the geometry of information. The probabilistic landscape over which state transitions for living systems are defined has a nontrivial curvature, since they are able to perform gradient descent. For example, in the context of predictive coding in the brain, this curvature can be interpreted as attention [65]. Living systems are organized such that their (physical) dynamics are also, at the same time, coextensive with trajectories over information landscapes. In other words, the FEP tells us that for an organism to follow Hamilton’s path of least action simply means that it must instantiate (embody and enact) a statistical model of its relation to its niche. ‘Inference’, then, is just the process whereby mutual information between organism and niche increases through generalized synchrony; it is not ‘inference’ in some strong propositional sense, but rather, a precise mathematical one (i.e., approximate Bayesian inference).
